# Evaluation of photobiomodulation effects on pain, edema, paresthesia, and bone regeneration after surgically assisted rapid maxillary expansion: Study protocol for a randomized, controlled, and double blind clinical trial

**DOI:** 10.1097/MD.0000000000017756

**Published:** 2019-11-27

**Authors:** Eduardo Vasques da Fonseca, Sandra Kalil Bussadori, Luiz Felipe Cabral da Silva Martinho, Maria Carolina de Souza Melo, Felipe Ledo de Andrade, Marcela Leticia Leal Gonçalves, Raquel Agnelli Mesquita-Ferrari, Anna Carolina Ratto Tempestini Horliana, Kristianne Porta Santos Fernandes

**Affiliations:** a Postgraduation Program in Biophotonics Applied to Health Sciences, Nove de Julho University - UNINOVE, Liberdade; b Department of Buccomaxillofacial Surgery and Traumatology of Mandaqui Hospital Complex, Santana, São Paulo, SP, Brazil.

**Keywords:** bone repair, edema, pain, paresthesia, photobiomodulation, randomized controlled trial, surgically assisted rapid maxillary expansion, transverse maxillary deficiency

## Abstract

**Background:**

Surgically assisted rapid maxillary expansion (SARME) generates an uncomfortable postoperative period accompanied by pain, edema, and paresthesia. There are few studies on the effect of photobiomodulation (PBM) after SARME and it was not possible to find studies on the efficacy of light emitted by diode (LED) after this type of intervention. The main objective of the study will be to evaluate the efficacy of PBM with LED in the control of pain, facial edema, paresthesia, and bone repair after SARME.

**Methods:**

A randomized, double-blind, placebo-controlled clinical trial involving 72 participants aged from 18 to 45 years, who search the Department of Buccomaxillofacial Surgery and Traumatology of Mandaqui Hospital Complex, will be conducted. Immediately after surgeries, the participant will be inserted into the placebo or LED group. In the LED group, the participants will receive PBM with an extraoral device (660 and 850 nm with 6 J per point) and an intraoral device (660 nm with 2 J per point) and in the control group the person in charge of the application will simulate the irradiation with the devices kept off. The applications will be in the immediate postoperative period, 1, 2, 7, 14, 30, 60, 90, and 120 days after the end of the surgeries, when the evaluations will also be performed. Facial measurements, extra and intraoral sensitivity, pain and bone repair will be evaluated. Secondarily, data regarding the occurrence of headache; otalgia; nausea; bruising; nasolacrimation; epistaxis; dysphagia; systemic and superficial temperature in the operated region; use of analgesics and anti-inflammatories; anxiety and impact of oral health on the participants’ quality of life will be computed.

**Discussion:**

Since PBM has shown positive effects on postoperative complications of other types of oral surgery and also has a positive effect on bone repair after maxillary disjunction, surgically assisted or not, it seems clear the need to evaluate its performance regarding pain, edema, and paresthesia after these surgeries.

**Trial registration:**

This protocol was registered in Clinical Trials platform (https://clinicaltrials.gov/) with the number NCT03814525, first published and last updated on January 24, 2019.

## 1 Introduction

The transverse maxillary deficiency, or maxillary narrowing, is usually accompanied by the presence of a high arched palate, reduction of the arch perimeter and negative space, crowding and dental rotation, and unilateral or bilateral posterior crossbite.^[1]^ A variety of surgical techniques, including surgically assisted rapid maxillary expansion (SARME) and Le Fort segmental osteotomies are the most suitable surgical options for the treatment of transverse maxillary deficiency,^[2]^ supplemented by the use of expanders. The intraoperative complications of SARME are not frequent, but postoperative complications are more common, especially edema, pain and paresthesia of infra-orbital nerves, which can reach up to 30% of cases.^[3–6]^

Although these complications occur frequently, there are few data on the effects of photobiomodulation (PBM) on these outcomes after SARME. As PBM has shown positive effects on the postoperative complications of other types of oral surgery^[7,8]^ and also acts positively on bone repair after maxillary disjunction, surgically assisted or not,^[9–11]^ there is a need to evaluate its performance regarding pain, edema, and paresthesia after these surgeries. Added to this, SARME reach large anatomical areas that involve intra and extraoral structures. Thus, it also seems clear that there is a need to evaluate the performance of light emitting diode (LED) sources that can reach larger areas in the same application and combine wavelengths that are absorbed by shallower and deeper tissues.

The main objectives of this study are to evaluate the effects of PBM on pain, edema, paresthesia, and bone repair in the postoperative period of surgical maxillary disjunction in patients of a Buccomaxillofacial Surgery Department of a public hospital in the State of São Paulo (Conjunto Hospitalar do Mandaqui), in different experimental periods. Secondarily, data regarding the occurrence of headache; otalgia; nausea; bruising; nasolacrimation; epistaxis; dysphagia; systemic and superficial temperature in the operated region; use of analgesics and anti-inflammatories; anxiety and impact of oral health on the participants’ quality of life will be computed.

## 2 Methods/design

### 2.1 Study design

This randomized, double-blind, placebo-controlled clinical study will follow Resolution 466/2012 of the National Health Council, Brazil. The project was evaluated and approved by the Research Ethics Committee of Universidade Nove de Julho (process number 03645518030015511) and by Conjunto Hospitalar do Mandaqui Hospital (process number 03645518000005551) and was registered in the Clinical Trials platform (https://clinicaltrials.gov/) with the number NCT03814525, first published and last updated on January 24, 2019. Surgeries and treatment with the PBM will be performed at the Mandaqui Hospital Complex in the city of São Paulo, Brazil, during the period from January 2, 2019 to January 30, 2021.

**Figure 1:**
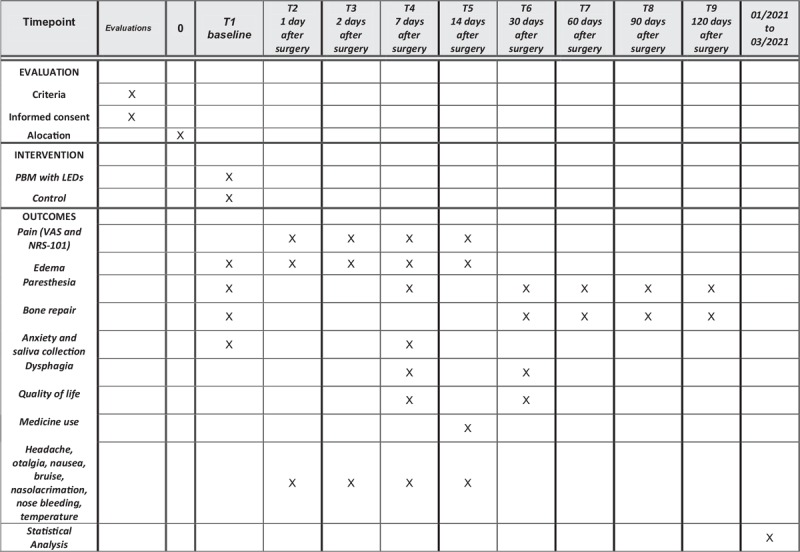
SPIRIT figure as recommended by the 2013 SPIRIT statement. SPIRIT = standard protocol items: recommendations for interventional trials.

The protocol is in accordance with the 2013 standard protocol items: recommendations for interventional trials (SPIRIT) statement. The SPIRIT checklist can be found as an additional file and Figure 1 is the SPIRIT figure. SPIRIT was developed to provide guidance in the form of a checklist of recommended items to include in a clinical trial protocol, to help improve its content and quality.

### 2.2 Participants

Participants of both genders, requiring surgical disjunction of the maxilla and that meet the eligibility criteria described below will be selected from the service of the Department of Buccomaxillofacial Surgery and Traumatology of Mandaqui Hospital Complex. After verbal explanation and reading about the procedures to be used in the study, patients who agree to participate will sign a free and informed consent term.

Inclusion criteria:

- Transverse maxillary deficiency greater than 5 mm with bilateral posterior crossbite and indication for surgical maxillary disjunction;
- Agree to participate in the study after reading and signing the term of consent for participation in clinical research;
- Aged between 18 and 45 years.

Exclusion criteria:

- Transverse maxilla deficiency, with unilateral posterior crossbite;
- Local or systemic alterations that contraindicate surgical intervention or hinder the postoperative period;
- Smokers;
- Display photosensitivity history;
- Possess systemic diseases, chronic pain, or neurological and psychiatric disorders;
- Have been using anti-inflammatories, analgesics, or bisphosphonates in the past 15 days;
- Pregnant;
- Breastfeeding;
- Participants who present any type of complication during surgery (hemorrhage, operative difficulty), as these cases will not be in the expected pattern for these surgeries. These data will not be part of the statistical analysis but will be described and discussed, as well as possible adverse effects.

### 2.3 Experimental groups

Participants will be divided in 2 groups:

- PBM group: the participants will receive PBM after surgery.
- Control group: participants will be attended to in the same way as the PBM group. The responsible for the application of the PBM will simulate the irradiations by positioning the devices in the same places as in the PBM group, but the equipment will be kept off. In order for the participant to not identify the group to which he/she belongs, the device activation sound (beep) will be recorded and switched on at the time of application.

### 2.4 Sample calculation

To determine the number of participants in each experimental group, a sample calculation based on the variability of the results of 3 articles that evaluated the main outcomes of this study in similar situations was performed.^[12–14]^ Using the unpaired *t* test method ^[15]^ the required sample would be 66 individuals, 33 per group. Calculations were performed using a significance level of 0.05 (implying a 5% Type I error and resulting in a 95% confidence interval analysis) and an absolute error of 5%. Considering a 10% loss, a loss of 3 participants per group should be predicted, thus, 72 patients will be recruited, 36 per group.

### 2.5 Randomization

To randomly distribute the subjects in the 2 experimental groups, a random sequence generator program (https://www.randomizer.org/tutorial/) will be used and the 6-member randomization option will be selected. Opaque envelopes will be identified with each number and inside it a sheet containing the information of the corresponding experimental group will be inserted according to the generated order. The envelopes will be sealed and will remain sealed in numerical order in a safe place until the time of the surgeries. The generation of the sequence and the preparation of the envelopes will be performed by a person who is not involved in the study.

### 2.6 Preoperative evaluation

#### 2.6.1 Calibration of examiners

The calibration process of the 2 researchers who will perform the pre and postoperative evaluations will consist of joint training exercises performed 3 times in 1 volunteer who will not be part of the experimental groups. The data will be discussed among these researchers to achieve an excellent level of agreement. Subsequently, each examiner will individually carry out the measures proposed in the study in 10 adult volunteers who will not be included in the sample, and the data obtained will be submitted to the intraclass correlation coefficient test. Once the agreement between the examiners is evaluated as excellent, the measurements will be performed on the sample participants.

#### 2.6.2 Edema evaluation

Using a flexible plastic millimeter tape (0.5 mm accuracy), 5 facial measurements will be taken, corresponding to the distances between the points listed below^[14]^

Line A – posterior tragus point to the most lateral point of the labial commissure;
Line B – tragus posterior point to pogonium;
Line C – tragus posterior point to the lateral corner of the eye;
Line D – lateral corner of eye to lowest point of jaw angle;
Line E – lower point of the angle of the mandible (gonio) to the middle of the nasal bone.

The measurements will be taken by 2 of the previously calibrated researchers. The values of the 5 measures (A, B, C, D, E) will be summed to obtain the data of each participant.

#### 2.6.3 Extra and intraoral sensitivity tests

The evaluation of extra and intraoral sensitivity will be performed in 6 regions: below the lower eyelid, cheek, nose wing, upper lip, oral buccal mucosa, and oral palatal mucosa on both sides. The tests will be performed sequentially, with a 2 to 3 minutes interval between each. The participant will receive instructions on the tests that will be initially performed on the back of his hand so he can become familiar. The test room will be prepared to provide a calm environment where the participant will be semi-seated and with eyes closed.^[13]^

#### 2.6.4 Evaluation of the sensation of light touch

For this evaluation, 20 mm fragments of 3-0, 4-0, 5-0, and 6-0 diameter mononylon suture threads (Ethicon; Johnson & Johnson, Cincinnati, OH) will be used. They will be fixed perpendicularly in a stainless steel holder of 10 cm in length. The participants’ response to the test will be classified (from 0 to 4 points) based on Table 1.^[13,16]^ In the case of lack of sensitivity, no points will be awarded, and a maximum of 4 points will be awarded for each side in each participant. The light touch sensation aims to evaluate the existence of an axonal neuropathy because it evaluates myelinated fibers type A alpha.^[13,16]^

**Table 1:**
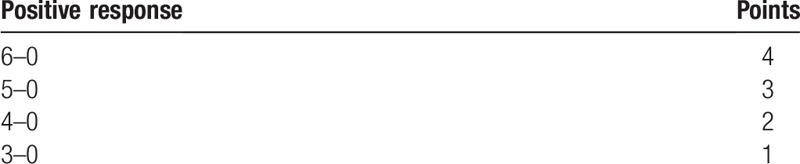
Attribution of points for positive response to the light touch test.

#### 2.6.5 Stinging sensation

This test will be performed using a very smoothly applied carpule syringe needle in the same areas described earlier on both sides of each participant. The determined response will be the perception of the touch of the needle, being recorded only as positive or negative, punctuating as: positive (1) and negative (0). This test aims to evaluate myelinated fibers type delta A and type C.^[13,16]^

#### 2.6.6 Static 2-point discrimination test

To perform this test, the same apparatus suggested by Gulses et al,^[13]^ aimed at evaluating the slow-adapting A beta myelinated fibers, will be made. Thus, a Biotone Dentsply color scale (Dentsply Sirona Brazil, São Paulo, Brazil) with 10 acrylic teeth will be used. Each tooth will receive, on the side face, 2 parallel holes separated by distances of 1 to 10 mm. Afterward, 0.8 mm stainless steel wires with a blunt tip will be inserted into these holes and fixed with acrylic resin. The crowns of the teeth will be marked with numbers that will identify the distance between the points. The test will be performed by applying the pair of tips very gently in the same areas described previously on both sides of each participant. Each pair of tips will be tested at once, starting with a random numbering. Then, the examiners will increase or decrease the space between the wires, testing the doubles of greater or lesser numbers as indicated by the initial response. The shortest distance in which the participant feels 2 tips will be considered.

#### 2.6.7 Dynamic 2-point discrimination test

Using the same device from the static test, now aiming to evaluate fast-adapting A beta myelinated fibers, a randomly chosen pair of tips will be moved from proximal to distal (within 1–2 cm), in the same anatomical regions as previously indicated, with as little pressure as possible. The participant will be asked to respond if they feel 1 or 2 tips. The numbering of the first pair of points will be chosen at random and then the order of use of the subsequent ones will be followed by increasing or decreasing their values. The shortest distance in which the participant feels 2 tips will be considered.

#### 2.6.8 Radiographic examination of the anterior region of the palatine suture

The optical density (OD) of the alveolar bone in the anterior region of the palatine suture will be the basis for evaluating bone repair after surgery.^[12]^ A periapical radiograph (of the upper incisors region with the radiographic technique of parallelism) of each participant using a 70 kVp RX transmitter will be obtained (Dabi Atlante Indústria Médico Odontológica LTDA, São Paulo, Brazil, Spectro 70X Selectronic). To standardize the radiographic shots in the different experimental periods, the focal length will be 30 cm and the exposure time will be 0.05 seconds. Five radiographic shots will be performed in different time frames, with all applicable biosafety system with individual protectors and lead apron. The intraoral positioner will be inserted with resistant molding material, silicone condensation (Optosil Xantopren – Heraeus Kulzer, Brazil, São Paulo), to obtain the impression of the edges of the lower incisor teeth that will guide the positioner in future shots. The radiographic films will be revealed in the same processor. The images will then be digitized and analyzed with the help of Image J software (National Institute of Health [NIH], Bethesda, Maryland).

To do this, an area in the anterior region between the upper central incisors will be selected for reading and counting the number of pixels of the scanned radiographic images. To obtain the delimitation of this area, the procedure described by Angeletti et al^[12]^ will be used. Initially, a straight line will be drawn on the alveolar bone crest between the crowns of the upper central incisors, which will be designated as the minor base (*b*). From this line, 2 lines (*h*) will be drawn parallel to the long axis of the right and left upper central incisors with a length of 15 mm. To join the lines h, another line will be drawn parallel to the smaller base which will be called the larger base (*B*), thus forming a trapezoid. The area and the number of pixels of each trapezoid will be recorded by the same professional in 3 different times with an interval of 15 days between each measurement. Then, the searcher will access the area values and number of acquired pixels and will calculate the average of these values. The trapezoidal area of each image will be calculated by the following formula: 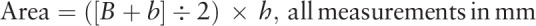


The OD in the region of the anterior palatine suture of each participant will be obtained by dividing the number of pixels by the trapezoid area.

#### 2.6.9 Anxiety analysis

The analysis of anxiety will be done through Beck inventory of anxiety that evaluates, by quantitative approximation, anxiety symptoms. The questionnaire contains 21 aspects that reflect somatically, cognitively, and affectively the characteristic symptoms of anxiety. The 21 statements describe the symptoms common in anxiety situations, which should be assessed by the participant with reference to himself or herself, in a 1-week period, on a 4-point scale, reflecting increasing levels of severity of each symptom, whose alternatives are:

(1) Absolutely not;
(2) Lightly: It did not bother me much;
(3) Moderately: It was very unpleasant, but I could bear it;
(4) Seriously: I could hardly bear it.

At the end, the items are summed and the total score can range from 0 to 63. All participants will respond to this questionnaire in the preoperative period and 7 days after surgery.

As anxiety may increase levels of cortisol and inflammatory cytokines adversely affecting the perception of pain and increasing recovery time after surgeries,^[17–21]^ its occurrence will also be evaluated by the immunoenzymatic dosage of inflammatory mediators (interleukin [IL]-1β, IL-6, tumor necrosis factor-α) and cortisol in saliva in the same periods of application of the questionnaire. To do so, participants will be asked to insert a cotton roller under the tongue for 2 to 3 minutes and then this cotton will be removed and placed in a special tube for later laboratory analysis by specific kits. The saliva remaining in the tubes will be discarded in an appropriate place.

### 2.7 Surgical procedure

#### 2.7.1 Planning and installation of expanders

Before the surgery, all participants will be molded and the respective models of plaster will be mounted in a semi-adjustable articulator, to perform measurements (between the canines and between the molars) and surgical planning. Later, simulation of surgical procedures will be performed in the models with the objective of achieving a stable occlusion and new measures will be performed (between the canines and between the molars). The measurements obtained before and after the simulation will be compared and the difference between them will give the exact measure of the maxillary expansion required.

After the planning stage, a Hyrax type dilator (Dentaurum 602-802; Ispringen, Germany) will be installed on the palate of each participant at least 24 hours before the surgeries. This expander will be fixed by means of orthodontic bands on the premolars and upper first molar of each participant and will not be activated. All expanders will be positioned by the same orthodontist.

#### 2.7.2 Surgical procedures (maxillar surgical disjunction [MSD])

The surgical procedures will be performed by 3 surgeons, specialized in maxillofacial surgery. The surgical material and the operative technique will be determined according to the protocol for MSD surgeries of the Maxillofacial Surgery and Traumatology Department of Conjunto Hospitalar do Mandaqui Hospital.

The following procedures will be made in every surgery:

- General anesthesia with nasotracheal intubation.
- Antisepsis of the operative field with alcoholic chlorhexidine 2% in the extraoral region and aqueous chlorhexidine 0.12% in the intraoral region.
- Placement of sterile surgical fields.
- Infiltrative anesthesia with 0.5% Bupivacaine with constricting vessel 1: 200,000 (10 mL) at the bottom of the vestibule in the region of the right and left first molar up to the upper canine region.
- Incision with scalpel blade number 15, extending from the first upper right molar to the first upper left molar.
- Mucoperiosteal syndesmotomy of the anterior region, exposing the anterior nasal spine and the pyriform aperture, followed for exposure of the canine and zygomatic pillars to the region of the pterygoid lamina.
- Delimitation of the bone area to be incised (5 mm above the dental apices) with a dry point compass.
- Osteotomy, with a sagittal saw, in the pyriform opening towards the zygomatic pillar. In the zygomatic pillar region, a reciprocating saw will be used to increase the osteotomy.
- Osteotomy of the maxillary pterygoid process with a curved chisel, 12 mm wide.
- Osteotomy of the nasal septum with a chisel.
- Vertical osteotomy of the anterior nasal spine to the interdental alveolar bone of the central incisors, with a 5 mm wide biselated chisel, directed to the medial sagittal line of the palate.
- Activation of Hyrax expander (Dentaurum). Surgical access suture with synthetic yarn 3.0 made with polyglactin 370 copolymer (30% glycolide and 70% L-lactide) and calcium stearate (vicryl, ethicon).
- All participants will receive the following medications in the immediate postoperative period: cefazolin 1 g (intravenous, EV) every 8 hours, dexamethasone 10 mg EV every 8 hours, dipyrone 1 g EV every 6 hours until hospital discharge days).

After hospital discharge, patients will be instructed to take amoxicillin 500 mg (oral tablet) every 8 hours for 7 days, dexamethasone 4 mg (oral tablet) every 8 hours for 3 days, dipyrone 500 mg (oral tablet) every 6 hours for 3 days and mouthwash with 5 mL of 0.1% chlorhexidine digluconate solution, 3× daily. At each return of participants, information will be requested on the amount of medicine ingested.

After the 7-day period, the participant will activate the Hyrax appliance, 1 mm per day (2 activations in the morning and 2 activations at night) until it reaches what was planned in the model surgery. After planned expansion, the device should be locked by the orthodontist for a period of 3 to 6 months.

### 2.8 Procedures to guarantee double blinding (patient and examiners) in the postoperative period

Once the suture is finished, the researcher responsible for applying PBM will remove and open the envelope containing the experimental group information in which the patient will be inserted and will proceed to the experiment. A single researcher will execute the PBM application and he/she will not perform any type of evaluation. The pre and postoperative evaluations will be done by 2 examiners who will not be aware of the group in which each patient is allocated. Participants will not be aware of whether or not they have received PBM, since the person responsible for the application will position the equipment in the irradiation locations in all participants and will only trigger the light when and where predicted in the specific experimental group. The characteristic sound of the device will be triggered by recording in the control group.

### 2.9 Photobiomodulation

PBM will be applied with extra and intraoral LED devices. The LED plates speed up the treatment as they deliver all the energy at once, having the advantage of radiating several points at the same time. The applications of both will occur in the following experimental periods after the completion of the surgeries: immediate postoperative, 1, 2, 7, 14, 30, 60, and 90 days.

#### 2.9.1 Extraoral PBM

Extraoral PBM will be applied with a mask (Cosmedical, São Paulo, Brazil) containing 57 red LEDs and 74 infrared LEDs, in the parameters described in Table 2 and in the periods described previously.

**Table 2:**
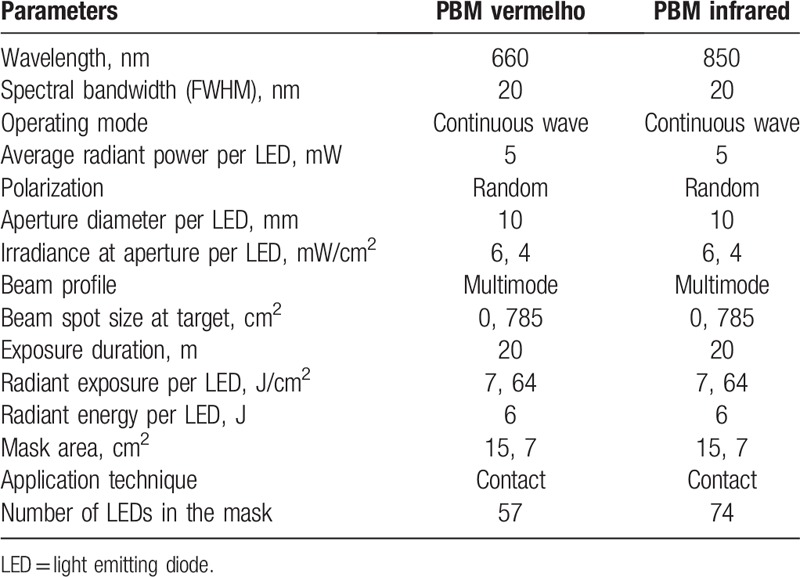
Extraoral LED mask dosimetric parameters.

#### 2.9.2 Intraoral PBM

Participants will receive intraoral LED with a specially designed oral device (Cosmedical) with the parameters described in Table 3, in the periods described above (the same as for extraoral irradiation).

**Table 3:**
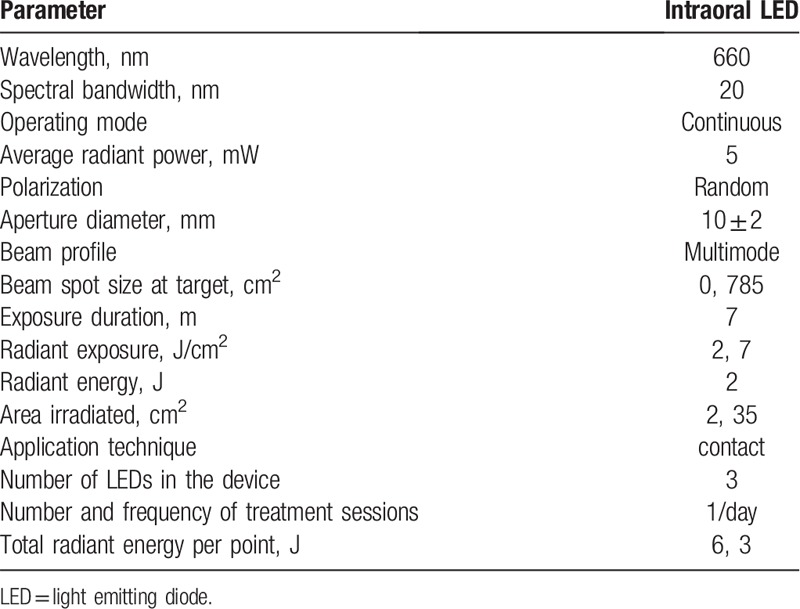
Dosimetric parameters of the intraoral LED device.

## 3 Outcome measures

All variables will be evaluated by the same 2 examiners, before and after surgery for periods of 1 to 120 days according to each variable. The primary outcomes of the study are: the occurrence and intensity of pain and edema (with evaluation periods of 1, 2, 7, and 14 days after surgery), the occurrence of paresthesia (with evaluation periods of 7, 30, 60, 90, and 120 days after surgery) and bone repair in the anterior palatine suture region (with evaluation periods of 30, 60, 90, and 120 days after surgery). The secondary outcomes of the study are: headache, otalgia, nausea, hematoma, nasolacrimation, nasal bleeding, and surface temperature (with evaluation periods of 1, 2, 7, and 14 days after surgery), dysphagia (with evaluation periods of 7 and 30 days after surgery), anxiety (7 days after surgery), quality of life (with periods of evaluations of 7 and 30 days after surgery), and use of analgesics and anti-inflammatory drugs on the 14th day. Information on gender (male/female), race, age (in years), and educational background (from illiterate to full graduate) will also be collected.^[22,23]^

### 3.1 Pain evaluation

Visual analog scales (VASs) are the most commonly used instruments for postoperative pain measurement after oral surgery using PBM postoperatively.^[22,24,25]^

On the other hand, the Numeric Pain Rating Scale (NRS)-101 scale is also being used to measure pain resulting from these surgeries^[26,27]^ and maybe more advantageous than that of VAS.

Both scales will be applied. VAS will be printed and the research participants will be instructed by the evaluators to mark a point in the 10 cm line, indicating the intensity of their pain after 1, 2, 7, and 14 days of surgeries. For NRS-101, the 2 examiners will ask participants to assign a number between 0 (no pain) and 100 (worst possible pain) that best represents the pain they are experiencing, in the same experimental periods.

### 3.2 Edema evaluation

To measure this outcome, the previous facial measures described in item 2.6.1., considered time 0 (T0), will be remade in the postoperative periods of 1, 2, 7, and 14 days, by the same 2 examiners who did the initial measurements. The edema of each participant will be defined as the difference between the sum of the measures of the 5 lines (of both sides) in each experimental period, and the sum of the same 5 lines before the surgery (T0).

### 3.3 Global sensitivity index

The sensitivity tests described above will be repeated in all participants, on both sides on postoperative days 7, 30, 60, 90, and 120. The results of the light touch and sting sensation tests will be added (maximum value 5 points/side/participant/period) and this value will be called qualitative sensitivity. On the other hand, the measurements obtained in the static 2-point discrimination test (item 2.6.5) and dynamic 2-point discrimination test (item 2.6.6) in each experimental period will be subtracted from those obtained in the respective tests in the preoperative evaluation and the value of the difference between these measures will be classified according to Table 4. The value of the sum of the classifications obtained in the static 2-point discrimination test (item 2.6.5) and dynamic 2-point discrimination test (item 2.6.6) in each experimental period will be denominated quantitative sensitivity (summing 0–10 points/side/participant/period).^[13,16]^

**Table 4:**
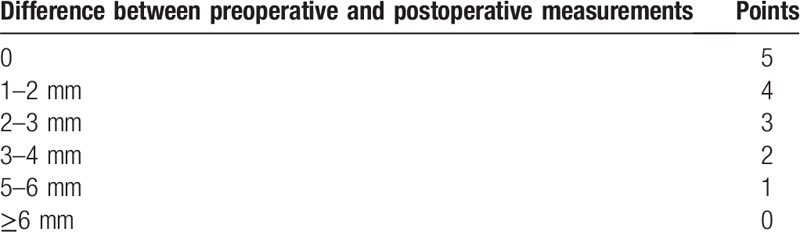
Classification for the differences obtained in the static 2-point discrimination test and dynamic 2-point discrimination test in the different periods.

Finally, the global sensitivity index (GSI) for each side of each participant in each experimental period will be calculated by adding the value of the qualitative sensitivity (sum of the light touch and stinging sensation) to the value of the quantitative sensitivity (sum of the classification obtained in the static 2-point discrimination test (item 2.6.5) and dynamic 2-point discrimination test (item 2.6.6)), its maximum value is equal to 15. The GSI will be classified according to Table 5, will be computed by hemi face in each experimental period, and may vary from normal sensitivity to reduced sensitivity.

**Table 5:**
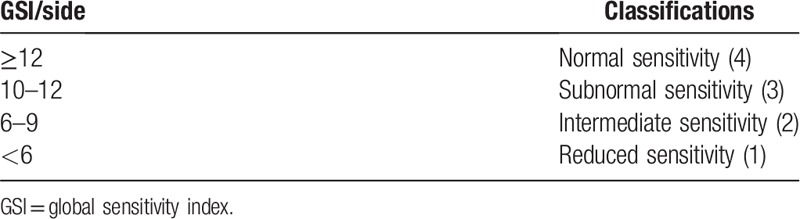
Classification for the differences obtained in GSI in the different periods.

### 3.4 Bone repair evaluation

Periapical radiographs will be obtained from each participant, as described previously, after 30, 60, 90, and 120 days of surgeries. The OD of the alveolar bone in the anterior region of the palatine suture, in each experimental period, will be calculated and compared with that obtained in the preoperative radiographic examination. Images will be scanned and analyzed with Image J software (NIH).

### 3.5 Secondary outcomes evaluation

The variables: local and systemic temperature, occurrence of headache, otalgia, nausea, hematoma, nasolacrimation, nasal bleeding, and the use of analgesics and anti-inflammatories will be evaluated in the periods of 1, 2, 7, and 14 days after surgery.

The temperature will be measured locally (on both sides) and systemically. The local measurements will be made with an infrared digital thermometer (model TL-612, Techline Comercial Importadora Exportadora e Serviços Ltda, São Paulo, Brazil) in the region of right and left cheeks and anterior nasal spine. The systemic temperature will be measured in the frontal region of the patient, in the median position, 3 cm above the glabella.

The presence of hematoma/ecchymosis will be evaluated by measuring the largest diameter of the colorimetric changes on the right and left side. The measure will be performed by the examiners, who will classify the occurrence of this result into 4 categories:

(1) none;
(2) larger diameter smaller than 4 cm;
(3) larger diameter between 4 and 10 cm, and
(4) larger diameter larger than 10 cm.^[28]^

For the analysis of anxiety, the same questionnaire described for the initial evaluation will be applied 7 days postoperatively. Saliva analysis will also be performed at these same times.

The variables: dysphagia and impact of the procedure on the quality of life of the participant will be evaluated in the periods of 7 and 30 days after surgery.

The evaluation of dysphagia will be performed after 7 and 30 days by questioning and classification on a numerical scale in which:

(0) total absence of dysphagia;
(1) dysphagia to solid foods;
(2) dysphagia to any liquid or solid food.

The impact of the surgical procedure on the participants’ quality of life will also be evaluated. The examiners will ask the participants to answer yes or no to the following 10 questions, after 7 and 30 days of the surgeries.^[29,30]^

(1) Are you maintaining your social activities normally?
(2) Are you working/studying normally?
(3) Are you maintaining your normal diet?
(4) Do you have difficulty swallowing because of the surgery?
(5) Do you have difficulty in tasting food?
(6) Can you chew on the sides?
(7) Do you have trouble sleeping because of the surgery?
(8) Did you have trouble speaking because of the surgery?
(9) Has your appearance changed because of the surgery?
(10) Do you feel sick from the surgery?

### 3.6 Statistical analysis

Initial descriptive analyses will be performed considering all variables measured in the study, both quantitative (mean and standard deviation) and qualitative (frequencies and percentages). Later, the appropriate statistical tests will be applied for each specific analysis. In all tests, the significance level of 5% probability or the corresponding *P*-value will be adopted. All analyses will be performed using the statistical software SAS for Windows, version 9.1.

## 4 Discussion

Two systematic reviews on the use of PBM after rapid maxillary disjunctions have recently been published.^[10,11]^ Both report that only 4 controlled and randomized clinical trials were conducted on the subject, and only one of them was performed with surgery.

Angeletti et al^[12]^ evaluated the effects of laser PBM on bone regeneration in the median region of the anterior suture after SARME. The authors observed that bone regeneration in the group receiving PBM was better in all experimental periods. Cepera et al^[31]^ evaluated the effects of laser PBM (GaAIAs, 780 nm, 40 mW, 10 J/cm^2^, 0.4 J per point) on bone regeneration and the success of orthodontic maxillary expansion using the Hyrax appliance. The authors concluded, after analysis of occlusal radiographs in 5 experimental periods, that the group that received PBM presented a greater expansion and better bone regeneration than the one that did not receive it. Garcia et al^[32]^ also studied the effects of PBM on repair of the medial palatine suture after maxillary orthodontic expansion. In the group that received PBM, the bone repair was superior. Finally, Ferreira et al,^[33]^ evaluated the effects of PBM on bone regeneration in the median palatine suture after rapid maxillary expansion using orthodontics (Hyrax type expander). Bone regeneration was assessed by measuring the OD in the images, and the authors also concluded that PBM positively influenced the bone regeneration of the medial palatine suture by accelerating the repair process.

Since PBM has shown positive effects on postoperative complications of other types of oral surgery^[7,8]^ and also has a positive effect on bone repair after maxillary disjunction, surgically assisted or not,^[10,11]^ it seems clear the need to evaluate its performance regarding pain, edema, and paresthesia after these surgeries. In addition, maxillary disjunction surgeries reach large anatomical areas involving intra and extraoral structures, so it also seems clear that there is a need to evaluate the performance of LED sources that can reach larger areas in the same application and combine wavelengths that are absorbed by superficial and deeper tissues.

## Author contributions

**Conceptualization:** Sandra Kalil Bussadori, Raquel Agnelli Mesquita-Ferrari, Kristianne Porta Santos Fernandes.

**Formal analysis:** Sandra Kalil Bussadori, Marcela Leticia Leal Gonçalves, Raquel Agnelli Mesquita-Ferrari, Anna Carolina Ratto Tempestini Horliana, Kristianne Porta Santos Fernandes.

**Investigation:** Eduardo Vasques da Fonseca, Luiz Felipe Cabral da Silva Martinho, Maria Carolina de Souza Melo, Felipe Ledo de Andrade.

**Methodology:** Eduardo Vasques da Fonseca, Kristianne Porta Santos Fernandes.

**Supervision:** Kristianne Porta Santos Fernandes.

**Writing – original draft:** Eduardo Vasques da Fonseca, Kristianne Porta Santos Fernandes.

**Writing – review and editing:** Sandra Kalil Bussadori, Raquel Agnelli Mesquita-Ferrari, Anna Carolina Ratto Tempestini Horliana, Kristianne Porta Santos Fernandes.
